# Isopropyl 3-oxo-2,3-dihydro-1,2-benzothia­zole-2-carboxyl­ate

**DOI:** 10.1107/S1600536811034209

**Published:** 2011-08-27

**Authors:** Xiang-hui Wang, Qiang Lin, Jian-xin Yang

**Affiliations:** a Institute of Environmental Science and Engineering, Kunming University of Science and Technology, Kunming 650093, People’s Republic of China; b Hainan Provincial Fine Chemical Engineering Center, Hainan University, Haikou 570228, People’s Republic of China; c College of Chemistry and Chemical Engineering, Hainan Normal University, Haikou 571100, People’s Republic of China; d Institute of Materials and Chemical Engineering, Hainan University, Haikou 570228, People’s Republic of China

## Abstract

The title compound, C_11_H_11_NO_3_S, was synthesized by the reaction of benzo[*d*]isothia­zol-3(2*H*)-one with isopropanol in toluene. The benzoisothia­zolone ring system is essentially planar, with a mean deviation of 0.018 (2) Å from the least–squares plane defined by the nine constituent atoms. In the crystal, mol­ecules are linked by weak inter­molecular C—H⋯O hydrogen bonds.

## Related literature

For background to the sythesis of benzoisothia­zolone derivatives, see: Davis (1972 ▶); Elgazwy & Abdel-Sattar (2003 ▶). For the biological activity of 1, 2–benzoisothia­zolone derivatives, see: Taubert *et al.* (2002 ▶). For structural studies of related alkyl 3-oxo-2,3-dihydro-1,2-benzothia­zole-2-carboxyl­ate derivatives, see: Wang *et al.* (2011*a* ▶,*b* ▶).
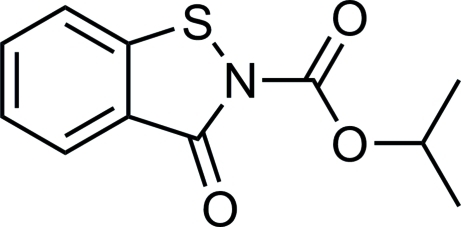


## Experimental

### 

#### Crystal data

C_11_H_11_NO_3_S
*M* *_r_* = 237.27
Orthorhombic, 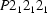

*a* = 4.6218 (19) Å
*b* = 11.621 (5) Å
*c* = 20.510 (9) Å
*V* = 1101.6 (8) Å^3^
*Z* = 4
Mo *K*α radiation
μ = 0.28 mm^−1^
*T* = 153 K
0.68 × 0.12 × 0.07 mm

#### Data collection

Rigaku AFC10/Saturn724+ diffractometer
Absorption correction: multi-scan (*ABSCOR*; Higashi, 1995 ▶) *T*_min_ = 0.830, *T*_max_ = 0.980
9436 measured reflections
2897 independent reflections
2245 reflections with *I* > 2σ(*I*)
*R*_int_ = 0.045

#### Refinement

*R*[*F* ^2^ > 2σ(*F* ^2^)] = 0.039
*wR*(*F* ^2^) = 0.078
*S* = 1.00
2897 reflections
147 parameters
H-atom parameters constrained
Δρ_max_ = 0.26 e Å^−3^
Δρ_min_ = −0.22 e Å^−3^
Absolute structure: Flack, (1983 ▶), 1150 Friedel pairs
Flack parameter: −0.02 (8)

### 

Data collection: *CrystalClear* (Rigaku, 2008 ▶); cell refinement: *CrystalClear*; data reduction: *CrystalClear*; program(s) used to solve structure: *SHELXS97* (Sheldrick, 2008 ▶); program(s) used to refine structure: *SHELXL97* (Sheldrick, 2008 ▶); molecular graphics: *SHELXTL* (Sheldrick, 2008 ▶); software used to prepare material for publication: *SHELXTL* and *publCIF* (Westrip, 2010 ▶).

## Supplementary Material

Crystal structure: contains datablock(s) I, global. DOI: 10.1107/S1600536811034209/lx2199sup1.cif

Structure factors: contains datablock(s) I. DOI: 10.1107/S1600536811034209/lx2199Isup2.hkl

Supplementary material file. DOI: 10.1107/S1600536811034209/lx2199Isup3.cml

Additional supplementary materials:  crystallographic information; 3D view; checkCIF report

## Figures and Tables

**Table 1:** Hydrogen-bond geometry (Å, °)

*D*—H⋯*A*	*D*—H	H⋯*A*	*D*⋯*A*	*D*—H⋯*A*
C2—H2⋯O1^i^	0.95	2.47	3.225 (3)	137

Symmetry code: (i) 
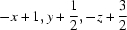
.

**Fig. 1.**
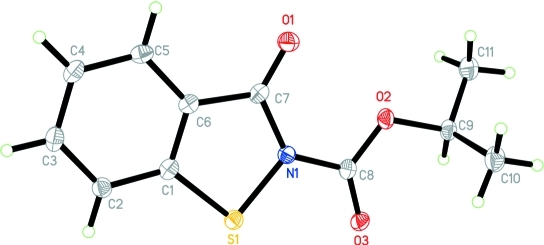
The molecular structure of the title compound with the atom numbering scheme. Displacement ellipsoids are drawn at the 30% probability level. H atoms are presented as a small spheres of arbitrary radius.

## supplementary crystallographic information

### Comment

1,2-benzoisothiazol-3(2*H*)-ones are a class of compounds with a wide spectrum of biological activities (Davis, 1972; Elgazwy & Abdel-Sattar, 2003). 1, 2-Benzisothiazolone derivatives have been reported to possess high antibacterial and antifungal activity (Taubert *et al.*, 2002). As a part of our ongoing study of the substituent effect on the solid state structures of alkyl 3-oxo-2,3-dihydro-1,2-benzothiazole-2-carboxylate analogues (Wang, *et al.*, 2011*a*,*b*), we report herein the crystal structure of the title compound.

The title compound crystallizes as the non–centrosymmetric space group *P*_21 21 21_ in spite of having no asymmetric C atoms. In the title molecule (Fig. 1), the benzoisothiazolone ring system is essentially planar, with a mean deviation of 0.018 (2) Å from the least–squares plane defined by the nine constituent atoms and the C8–C2–C9–C11 torsion angle is 156.63 (18)°. The crystal packing  is stabilized by weak intermolecular C—H···O hydrogen bonds between a benzene H atom and the O atom of the carbonyl group (Table 1; C2—H2···O1^i^).

### Experimental

A solution (20 ml) containing benzo[*d*]isothiazol-3(2*H*)-one (1.51 g, 0.01 mol) was added dropwise to a solution of isopropanol (0.61 g, 0.01 mol) and bis(triehloromethyl)Carbonate in toluene (20 ml) under stirring on an ice-water bath. The reaction mixture was stirred at room temperature for 4.5 h to afford the title compound (1.72 g, yield 72.6%). Single crystals suitable for X-ray measurements were obtained by recrystallization of the title compound from cyclohexane at room temperature.

### Refinement

The H atoms were placed at calculated positions and refined in riding mode, with the carrier atom–H distances = 0.95 Å for aryl, 0.99Å for methylene, 0.98 Å for the methyl. The *U*iso values were constrained to be 1.5*U*eq of the carrier atom for the methyl H atoms and 1.2*U*eq for the remaining H atoms.

### Crystal data

**Table:**

C_11_H_11_NO_3_S	*F*(000) = 496
*M**_r_* = 237.27	*D*_x_ = 1.431 Mg m^−^^3^
Orthorhombic, *P*2_1_2_1_2_1_	Mo *K*α radiation, λ = 0.71073 Å
Hall symbol: P 2ac 2ab	Cell parameters from 3268 reflections
*a* = 4.6218 (19) Å	θ = 2.7–29.1°
*b* = 11.621 (5) Å	µ = 0.28 mm^−^^1^
*c* = 20.510 (9) Å	*T* = 153 K
*V* = 1101.6 (8) Å^3^	Prism, colorless
*Z* = 4	0.68 × 0.12 × 0.07 mm

### Data collection

**Table:**

AFC10/Saturn724+ diffractometer	2897 independent reflections
Radiation source: Rotating Anode	2245 reflections with *I* > 2σ(*I*)
graphite	*R*_int_ = 0.045
Detector resolution: 28.5714 pixels mm^-1^	θ_max_ = 29.1°, θ_min_ = 3.5°
phi and ω scans	*h* = −6→6
Absorption correction: multi-scan (*ABSCOR*; Higashi, 1995)	*k* = −14→15
*T*_min_ = 0.830, *T*_max_ = 0.980	*l* = −28→26
9436 measured reflections	

### Refinement

**Table:**

Refinement on *F*^2^	Secondary atom site location: difference Fourier map
Least-squares matrix: full	Hydrogen site location: difference Fourier map
*R*[*F*^2^ > 2σ(*F*^2^)] = 0.039	H-atom parameters constrained
*wR*(*F*^2^) = 0.078	*w* = 1/[σ^2^(*F*_o_^2^) + (0.0289*P*)^2^ + 0.116*P*] where *P* = (*F*_o_^2^ + 2*F*_c_^2^)/3
*S* = 1.00	(Δ/σ)_max_ < 0.001
2897 reflections	Δρ_max_ = 0.26 e Å^−^^3^
147 parameters	Δρ_min_ = −0.22 e Å^−^^3^
0 restraints	Absolute structure: Flack, (1983), 1150 Friedel pairs
Primary atom site location: structure-invariant direct methods	Flack parameter: −0.02 (8)

### Special details

**Table:**

Geometry. All e.s.d.'s (except the e.s.d. in the dihedral angle between two l.s. planes) are estimated using the full covariance matrix. The cell e.s.d.'s are taken into account individually in the estimation of e.s.d.'s in distances, angles and torsion angles; correlations between e.s.d.'s in cell parameters are only used when they are defined by crystal symmetry. An approximate (isotropic) treatment of cell e.s.d.'s is used for estimating e.s.d.'s involving l.s. planes.
Refinement. Refinement of *F*^2^ against ALL reflections. The weighted *R*-factor *wR* and goodness of fit *S* are based on *F*^2^, conventional *R*-factors *R* are based on *F*, with *F* set to zero for negative *F*^2^. The threshold expression of *F*^2^ > σ(*F*^2^) is used only for calculating *R*-factors(gt) *etc*. and is not relevant to the choice of reflections for refinement. *R*-factors based on *F*^2^ are statistically about twice as large as those based on *F*, and *R*- factors based on ALL data will be even larger.

### Fractional atomic coordinates and isotropic or equivalent isotropic displacement parameters (Å^2^)

**Table:**

	*x*	*y*	*z*	*U*_iso_*/*U*_eq_	
S1	0.56113 (12)	0.76406 (4)	0.72970 (2)	0.02649 (13)	
O1	0.3920 (3)	0.45795 (12)	0.67074 (7)	0.0339 (4)	
O2	0.7876 (3)	0.55858 (12)	0.59264 (7)	0.0289 (3)	
O3	0.9282 (3)	0.73722 (11)	0.62359 (6)	0.0303 (3)	
N1	0.5743 (4)	0.64449 (13)	0.67914 (8)	0.0231 (4)	
C1	0.3073 (4)	0.69457 (17)	0.77902 (10)	0.0258 (5)	
C2	0.1822 (4)	0.73839 (18)	0.83559 (10)	0.0285 (5)	
H2	0.2327	0.8124	0.8516	0.034*	
C3	−0.0175 (5)	0.67062 (18)	0.86754 (11)	0.0333 (6)	
H3	−0.1053	0.6986	0.9063	0.040*	
C4	−0.0940 (5)	0.56146 (18)	0.84420 (10)	0.0319 (5)	
H4	−0.2360	0.5175	0.8666	0.038*	
C5	0.0350 (5)	0.51759 (17)	0.78915 (10)	0.0283 (5)	
H5	−0.0128	0.4428	0.7738	0.034*	
C6	0.2375 (5)	0.58518 (16)	0.75632 (10)	0.0236 (4)	
C7	0.3987 (4)	0.55027 (18)	0.69814 (10)	0.0246 (4)	
C8	0.7810 (5)	0.65258 (17)	0.62952 (10)	0.0248 (5)	
C9	0.9960 (4)	0.56000 (18)	0.53842 (10)	0.0293 (5)	
H9	1.1790	0.5981	0.5529	0.035*	
C10	0.8682 (6)	0.62614 (19)	0.48198 (11)	0.0400 (6)	
H10A	0.6858	0.5902	0.4687	0.048*	
H10B	1.0041	0.6254	0.4453	0.048*	
H10C	0.8318	0.7058	0.4954	0.048*	
C11	1.0554 (6)	0.43512 (19)	0.52321 (12)	0.0432 (6)	
H11A	1.1449	0.3982	0.5611	0.052*	
H11B	1.1866	0.4299	0.4858	0.052*	
H11C	0.8731	0.3961	0.5128	0.052*	

### Atomic displacement parameters (Å^2^)

**Table:**

	*U*^11^	*U*^22^	*U*^33^	*U*^12^	*U*^13^	*U*^23^
S1	0.0236 (2)	0.0281 (3)	0.0277 (2)	−0.0018 (2)	0.0024 (2)	−0.0002 (2)
O1	0.0339 (9)	0.0294 (8)	0.0386 (9)	−0.0041 (7)	0.0053 (8)	−0.0039 (7)
O2	0.0272 (8)	0.0338 (8)	0.0256 (8)	−0.0021 (7)	0.0072 (6)	−0.0037 (7)
O3	0.0312 (8)	0.0314 (8)	0.0284 (7)	−0.0096 (8)	0.0033 (7)	−0.0001 (6)
N1	0.0192 (8)	0.0240 (8)	0.0261 (9)	−0.0014 (8)	0.0021 (8)	−0.0011 (7)
C1	0.0184 (10)	0.0304 (11)	0.0286 (11)	0.0032 (8)	−0.0010 (9)	0.0056 (9)
C2	0.0284 (11)	0.0292 (11)	0.0277 (10)	−0.0002 (10)	0.0001 (9)	−0.0004 (9)
C3	0.0330 (14)	0.0401 (13)	0.0267 (11)	0.0055 (10)	0.0060 (9)	0.0043 (9)
C4	0.0271 (12)	0.0346 (12)	0.0341 (12)	0.0014 (11)	0.0050 (10)	0.0105 (10)
C5	0.0234 (11)	0.0239 (11)	0.0376 (12)	0.0004 (9)	−0.0024 (10)	0.0040 (9)
C6	0.0181 (10)	0.0255 (11)	0.0273 (11)	0.0031 (8)	−0.0042 (8)	0.0048 (8)
C7	0.0172 (10)	0.0290 (11)	0.0275 (10)	−0.0003 (9)	−0.0023 (9)	0.0047 (8)
C8	0.0218 (11)	0.0313 (12)	0.0215 (10)	0.0023 (9)	−0.0029 (9)	0.0028 (9)
C9	0.0262 (13)	0.0373 (12)	0.0244 (10)	−0.0021 (10)	0.0055 (9)	−0.0009 (9)
C10	0.0405 (16)	0.0507 (14)	0.0289 (12)	−0.0036 (12)	−0.0017 (11)	0.0002 (11)
C11	0.0464 (15)	0.0454 (13)	0.0377 (13)	0.0011 (14)	0.0115 (13)	−0.0039 (11)

### Geometric parameters (Å, °)

**Table:**

S1—N1	1.7349 (17)	C4—C5	1.375 (3)
S1—C1	1.747 (2)	C4—H4	0.9500
O1—C7	1.212 (2)	C5—C6	1.395 (3)
O2—C8	1.329 (2)	C5—H5	0.9500
O2—C9	1.472 (2)	C6—C7	1.464 (3)
O3—C8	1.202 (2)	C9—C11	1.509 (3)
N1—C8	1.399 (3)	C9—C10	1.510 (3)
N1—C7	1.418 (3)	C9—H9	1.0000
C1—C6	1.392 (3)	C10—H10A	0.9800
C1—C2	1.393 (3)	C10—H10B	0.9800
C2—C3	1.379 (3)	C10—H10C	0.9800
C2—H2	0.9500	C11—H11A	0.9800
C3—C4	1.401 (3)	C11—H11B	0.9800
C3—H3	0.9500	C11—H11C	0.9800
			
N1—S1—C1	89.97 (9)	O1—C7—C6	127.63 (19)
C8—O2—C9	115.85 (16)	N1—C7—C6	107.53 (17)
C8—N1—C7	130.00 (17)	O3—C8—O2	127.0 (2)
C8—N1—S1	113.89 (13)	O3—C8—N1	121.00 (19)
C7—N1—S1	115.74 (14)	O2—C8—N1	112.00 (17)
C6—C1—C2	121.1 (2)	O2—C9—C11	105.33 (17)
C6—C1—S1	112.58 (15)	O2—C9—C10	109.20 (17)
C2—C1—S1	126.31 (17)	C11—C9—C10	113.71 (19)
C3—C2—C1	117.7 (2)	O2—C9—H9	109.5
C3—C2—H2	121.1	C11—C9—H9	109.5
C1—C2—H2	121.1	C10—C9—H9	109.5
C2—C3—C4	121.6 (2)	C9—C10—H10A	109.5
C2—C3—H3	119.2	C9—C10—H10B	109.5
C4—C3—H3	119.2	H10A—C10—H10B	109.5
C5—C4—C3	120.5 (2)	C9—C10—H10C	109.5
C5—C4—H4	119.8	H10A—C10—H10C	109.5
C3—C4—H4	119.8	H10B—C10—H10C	109.5
C4—C5—C6	118.6 (2)	C9—C11—H11A	109.5
C4—C5—H5	120.7	C9—C11—H11B	109.5
C6—C5—H5	120.7	H11A—C11—H11B	109.5
C1—C6—C5	120.5 (2)	C9—C11—H11C	109.5
C1—C6—C7	114.09 (18)	H11A—C11—H11C	109.5
C5—C6—C7	125.36 (18)	H11B—C11—H11C	109.5
O1—C7—N1	124.83 (19)		
			
C1—S1—N1—C8	−175.20 (15)	S1—N1—C7—O1	−175.99 (17)
C1—S1—N1—C7	−1.47 (16)	C8—N1—C7—C6	175.40 (19)
N1—S1—C1—C6	−0.54 (15)	S1—N1—C7—C6	2.9 (2)
N1—S1—C1—C2	178.93 (19)	C1—C6—C7—O1	175.6 (2)
C6—C1—C2—C3	−1.3 (3)	C5—C6—C7—O1	−2.9 (3)
S1—C1—C2—C3	179.25 (17)	C1—C6—C7—N1	−3.3 (2)
C1—C2—C3—C4	−0.2 (3)	C5—C6—C7—N1	178.28 (19)
C2—C3—C4—C5	1.6 (3)	C9—O2—C8—O3	−0.8 (3)
C3—C4—C5—C6	−1.6 (3)	C9—O2—C8—N1	179.12 (16)
C2—C1—C6—C5	1.4 (3)	C7—N1—C8—O3	−173.8 (2)
S1—C1—C6—C5	−179.12 (16)	S1—N1—C8—O3	−1.2 (3)
C2—C1—C6—C7	−177.15 (18)	C7—N1—C8—O2	6.3 (3)
S1—C1—C6—C7	2.3 (2)	S1—N1—C8—O2	178.90 (13)
C4—C5—C6—C1	0.1 (3)	C8—O2—C9—C11	156.63 (18)
C4—C5—C6—C7	178.46 (19)	C8—O2—C9—C10	−80.9 (2)
C8—N1—C7—O1	−3.5 (3)		

### Hydrogen-bond geometry (Å, °)

**Table:**

*D*—H···*A*	*D*—H	H···*A*	*D*···*A*	*D*—H···*A*
C2—H2···O1^i^	0.95	2.47	3.225 (3)	137.

Symmetry codes: (i) −*x*+1, *y*+1/2, −*z*+3/2.
